# Sordariomycetes Taxa Associated with *Dracaena* in Karst Outcrops: Two Novel Species and Five New Host Records from Thailand

**DOI:** 10.3390/jof12030168

**Published:** 2026-02-26

**Authors:** Napalai Chaiwan, Saowaluck Tibpromma, Samantha C. Karunarathna, Dhanushka N. Wanasinghe, Kevin D. Hyde, Nakarin Suwannarach, Ruvishika S. Jayawardena, Itthayakorn Promputtha

**Affiliations:** 1 Office of Research Administration, Chiang Mai University, Chiang Mai 50200, Thailand; napalai.c@cmu.ac.th (N.C.); nakarin.su@cmu.ac.th (N.S.); 2 Department of Biology, Faculty of Science, Chiang Mai University, Chiang Mai 50200, Thailand; 3 Center for Yunnan Plateau Biological Resources Protection and Utilization & Yunnan International Joint Laboratory of Fungal Sustainable Utilization in South and Southeast Asia, College of Biology and Food Engineering, Qujing Normal University, Qujing 655099, China; saowaluckfai@gmail.com (S.T.); samanthakarunarathna@gmail.com (S.C.K.); 4 Center for Mountain Futures, Kunming Institute of Botany, Chinese Academy of Sciences, Honghe 654400, China; dnadeeshan@gmail.com; 5 Department of Soil Science, College of Food and Agriculture Sciences, King Saud University, P.O. Box 145111, Riyadh 11362, Saudi Arabia; 6 Center of Excellence in Fungal Research, Mae Fah Luang University, Chiang Rai 57100, Thailand; kdhyde3@gmail.com; 7 Mushroom Research Foundation, 128 M.3 Ban Pa Deng T. Pa Pae, A. Mae Taeng, Chiang Mai 50150, Thailand; 8 School of Science, Mae Fah Luang University, Chiang Rai 57100, Thailand; 9 Center of Excellence in Microbial Diversity and Sustainable Utilization, Chiang Mai University, Chiang Mai 50200, Thailand; 10 Department of Biology, College of Science, Kyung Hee University, Seoul Campus, Seoul 02447, Republic of Korea

**Keywords:** 2 new species, Asparagaceae, biodiversity, molecular phylogeny, morphology, multi-loci phylogenetic analysis

## Abstract

Currently, our understanding of the fungi associated with *Dracaena* species is limited. There is a clear need for more comprehensive information, especially in the context of Thailand. In our study, we collected dead *Dracaena* leaves with fungal structures from limestone outcrops in seven Thai provinces: Chiang Mai, Kanchanaburi, Krabi, Nakhon Si Thammarat, Ratchaburi, Songkhla, and Tak. The fungi in these samples were isolated and identified using a combination of morphological characteristics and a multi-loci phylogeny (ACT, CHS-1, GAPDH, ITS, LSU, and TUB2). We are thrilled to introduce seven taxa belonging to four families within three orders (Chaetosphaeriales, Glomerellales, and Xylariales). Our detailed morphological descriptions and updated phylogenetic trees of two new species (*Zygosporium dracaenae*, and *Z. dracaenicola*) and five new host/geographical records (*Colletotrichum dracaenophilum*, *C. gigasporum*, *C. truncatum*, *Malaysiasca phaii,* and *Neoleptosporella camporesiana*) represent a significant step forward in our understanding of this field.

## 1. Introduction

Limestone outcrops are unique landscapes formed by dissolved soluble rocks in tropical regions [1]. These geological formations result from the rocky desertification of carbonate bedrock, characterized by calcium-rich, phosphorus-deficient, slightly alkaline soils, along with soil erosion and land degradation, making vegetation recovery challenging [2]. Limestone outcrops are rich in biodiversity and host numerous native plant species with ornamental and medicinal uses [3,4]. In Thailand, limestone outcrops are most prominent in high mountain regions, with the largest massif located in Chiang Mai (Doi Chiang Dao). They are also commonly found in Saraburi and the southern provinces such as Krabi and Surat Thani [5,6]. These outcrops in Thailand support a unique array of plant species distinct from other habitats, yet the fungal diversity associated with limestone in the region remains largely unexplored.

*Dracaena* is widely distributed across Asia and thrives in various habitats, including karst limestone outcrops, sunlit areas, cliffs, and escarpments [7,8,9]. Belonging to the Asparagaceae family, *Dracaena* is a monocotyledon genus that includes between 750 and 1200 species [10]. *Dracaena* species are found across diverse regions, including Africa, Australia, Central and Northwestern South America, Cuba, Hawaii, Mexico, Macaronesia, Madagascar, Micronesia, Indian Ocean islands, South Asia, Southeast Asia, and the Arabian Peninsula [11].

*Dracaena* species are commonly used as ornamental and medicinal plants [12] in Europe and Canada. *Dracaena marginata*, a popular houseplant, is a significant ornamental species known for reducing formaldehyde levels in indoor air [13]. Various fungi, particularly Ascomycota, are associated with *Dracaena* as an endophytes, pathogens or saprobes [14,15,16,17,18,19]. The fungi linked to *Dracaena* exhibits remarkable diversity [14]. For example, Thongkantha et al. (2008) [14] studied fungi from *Dracaena* and *Pandanus* in Thailand and documented 127 saprobic fungi, including 40 ascomycetes, one basidiomycete, and 86 asexual taxa; eight of these ascomycetes and three asexual taxa were newly discovered. Since 2010, reports of microfungi on *Dracaena* using both morphological and phylogenetic approaches have been limited [15,18,20].

The study of Sordariomycetes taxa associated with *Dracaena* is particularly significant, highlighting the crucial role of this diverse group in various ecological and practical contexts. Sordariomycetes, known for their wide distribution and morphological diversity, serve important functions in ecosystems such as plant and human pathogens, saprobes, endophytes, and fungicolous species, contributing to ecological balance and biodiversity [21]. Beyond their ecological importance, Sordariomycetes are also noted for producing a wide array of chemically distinct and varied metabolites with numerous applications in key fields, underscoring their potential for novel discoveries [22]. The expanding habitats of *Dracaena* have led to increased fungal diseases, particularly anthracnose caused by *Colletotrichum* species, underscoring the urgent need for in-depth studies in this area [17,18]. Despite these growing concerns, research on the diversity and ecology of pathogens and endophytes associated with *Dracaena* remains limited. This gap emphasizes the need to focus on Sordariomycetes linked to *Dracaena* to enhance our understanding of their ecological roles, disease management potential, and the exploration of beneficial metabolites they may offer.

This study presents an in-depth investigation of microfungi associated with *Dracaena* species in Thailand, emphasizing the vital role of taxonomy in unraveling fungal diversity and ecology. Through a combined approach of morphological assessment and molecular data analysis, we aim to comprehensively identify the species involved. Our findings enhance the understanding of fungal biodiversity linked to *Dracaena* in Thailand and underscore the importance of a robust taxonomic framework for exploring plant-microbe interactions. By documenting various Ascomycota species within this unique ecological setting, this study makes a significant contribution to fungal systematics.

## 2. Materials and Methods

### 2.1. Sample Collection, Specimen Examination and Isolation of Fungi

Specimens were collected during both the dry season (February and October) and the rainy seasons (May–September) from seven provinces in Thailand: Chiang Mai, Kanchanaburi, Krabi, Nakhon Si Thammarat, Ratchaburi, Songkhla, and Tak. Each sample was stored in an envelope or plastic bag, and important information was recorded [23]. The sample was transported to the laboratory for observation and fungal isolation.

Micro-morphological structures were examined using a Nikon ECLIPSE 80i compound light microscope (Nikon Corporation, Tokyo, Japan) and photographed with a Canon EOS 600D camera (Canon Inc., Tokyo, Japan) attached to the microscope. Measurements were made using Tarosoft^®^ Image Framework (Tarosoft Co., Ltd., Bangkok, Thailand), and figures were edited with Adobe Photoshop CS6 Extended v.10.0 (Adobe Systems, USA). Fungi on dead leaves were isolated using single spores, as outlined by Senanayake [24]. Germinating spores were aseptically transferred to fresh potato dextrose agar (PDA) plates (potatoes 85%; dextrose 8%; and agar 6%) and incubated at 25 °C. Cultures were grown for 14–28 days, during which colony color, texture, and other morphological characteristics were recorded. Representative cultures were used for morphological examination and DNA extraction and sequencing, and subsequently deposited in recognized culture collections.

Specimens and living cultures were deposited in the Fungarium of Mae Fah Luang University (MFLU) and the Culture Collection of Mae Fah Luang University (MFLUCC), Chiang Rai, Thailand. Faces of Fungi and Index Fungorum numbers were assigned following the protocols outlined by Jayasiri et al. [25] and Index Fungorum [26]. New taxa were established in accordance with the guidelines recommended by Jeewon & Hyde [27], Chethana [28], and Jayawardena et al. [29].

### 2.2. DNA Extraction, PCR Amplification and Sequencing

DNA extraction, amplification, and sequencing were conducted following the method outlined by Dissanayake [30]. Genes were amplified using universal primers (Table 1), and the PCR products were purified and sequenced with the same primers. Amplification reactions were performed as described by Dissanayake [30]. The quality of the PCR products was assessed using 1% agarose gel electrophoresis stained with ethidium bromide. The purified PCR products were then sent for sequencing to Sangon Biotech in Kunming, China.

### 2.3. Phylogenetic Analysis

All sequences from each strain were used for BLAST searches in the GenBank nucleotide database (https://blast.ncbi.nlm.nih.gov/Blast.cgi?PAGE_TYPE=BlastSearch&fbclid=IwAR09XiMGK90VkdxQYAbtT19OEikz4ApimtpnDVJTHOETr9BG8NxMDZHDkRQ, accessed on 28 August 2025) to identify their closest taxonomic matches. The phylogenetic positions of Sordariomycetes isolates were initially assessed using sequence data from *ACT*, *CHS*, *GAPDH*, ITS, LSU and *TUB* genes. Species-level identifications were then refined using multi-loci phylogeny based on individual and combined gene regions. Reference sequences and outgroups for each fungal group were selected from recent literature and GenBank. Individual genomic region datasets were aligned using the MAFFT version 7.221 server (http://mafft.cbrc.jp/alignment/software/, accessed on 28 August 2025). Phylogenetic trees were constructed using Maximum Likelihood (ML) and Maximum Parsimony (MP), with bootstrap values of 60% or greater provided at each node.

Maximum parsimony analysis (MP) was conducted using the heuristic search option in PAUP (Phylogenetic Analysis Using Parsimony) v. 4.0b10 with the following parameters: characters were treated as unordered with equal weight, random taxon addition was used, and branch swapping was performed with the tree bisection-reconnection (TBR) algorithm, collapsing branches with a maximum length of zero. Alignment gaps were considered as missing characters in the analysis of the combined dataset, particularly in relatively conserved regions. Trees were inferred using heuristic search with 1000 random sequence additions and a maximum of 1000 trees. Descriptive tree statistics, including tree length (TL), consistency index (CI), retention index (RI), relative consistency index (RC), and homoplasy index (HI), were calculated for the generated trees. The Kishino-Hasegawa tests [36] were performed to assess whether the trees differed significantly.

Phylogenetic analyses were conducted using maximum likelihood (ML) trees generated with RAxML-HPC2 on XSEDE (8.2.8) [37,38] via the CIPRES Science Gateway platform Miller [39], employing the GTR + I + G model of evolution.

Bayesian analysis was performed with MrBayes v. 3.1.2 [40], utilizing GTR + I + G for the command and evaluating Bayesian Posterior Probabilities (BYPP) [41] using Markov chain Monte Carlo sampling. Six simultaneous Markov chains were run for 2,000,000 generations, with trees sampled every 200th generation. The distribution of log-likelihood scores was assessed using Tracer 1.4 [42] to determine the stationary phase and whether additional runs were necessary for convergence. The first 10% of trees were discarded, and the remaining 90% were used to calculate the posterior probabilities of the majority rule consensus tree, with BYPP values greater than 0.95 indicated at each node. Phylogenetic trees were visualized and annotated using Treeview, version 32 [43] and formatted with PowerPoint 2019 (Microsoft Corporation, WA, USA).

### 2.4. Genealogical Concordance Phylogenetic Species Recognition Analysis

The Genealogical Concordance Phylogenetic Species Recognition (GCPSR) model was used to analyze related species by identifying significant recombination events and assessing recombination levels within closely related species. This model supports the significance of new species within a closely related clade. The Pairwise Homoplasy Index (PHI) test, which assesses whether multiple gene phylogenies are concordant between species or discordant due to recombination and mutations within species, was performed using SplitsTree4 as described by Quaedvlieg [44]. The test employed a concatenated locus dataset to determine recombination levels among phylogenetically closely related species. A PHI value below 0.05 (Φw < 0.05) indicates significant recombination within the dataset, suggesting no significant differences among related species. Conversely, a PHI value above 0.05 (Φw > 0.05) suggests no significant recombination, indicating differences among related species at the group level. The model was applied to analyze the new species and its closely related species, and relationships between these species were visualized by constructing a split graph using both LogDet transformation and split decomposition options.

## 3. Results

### 3.1. Taxonomy and Phylogeny

#### 3.1.1. Chaetosphaeriales Huhndorf, A.N. Mill. & F.A. Fernández

Huhndorf [45] established Chaetosphaeriales to accommodate *Chaetosphaeriaceae*. Hyde [46] recognized six families and 119 genera within this order. Phukhamsakda et al. [47] introduced *Neoleptosporella* as a new lineage within *Chaetosphaeriaceae*, and later, Zhang et al. [48] proposed *Neoleptosporellaceae* to accommodate species of *Neoleptosporella*.

This study differs from previous work by reporting from *Dracaena* sp. in Tak Province, Thailand, representing a novel host and locality for the genus, which was previously known only from *Clematis subumbellata* (*Ranunculaceae*) in Chiang Rai. This finding significantly contributes to our understanding of the ecological diversity of *Chaetosphaeriaceae* within Chaetosphaeriales, highlighting the importance of this research in the field of mycology.

#### 3.1.2. *Neoleptosporellaceae* J.F. Zhang, Y.Y. Chen & Jian K. Liu

Zhang [48] established *Neoleptosporellaceae* to support the *Neoleptosporella* species. Although *Neoleptosporellaceae* is phylogenetically closely related to *Helminthosphaeriaceae*, it is distinctly differentiated from this latter by its stromatic ascomata, which can be globose or immersed. In *Helminthosphaeriaceae*, ascospores are often ellipsoid or fusoid and frequently have septation. Also, your representatives are associated with decaying plant material and sometimes exhibit dark pigmentation in their structures. In contrast, *Neoleptosporellaceae* forms pycnidia (asexual structures) and perithecia (sexual structures). Spores in this family are usually fusiform, hyaline (light-colored), and exhibit a more diverse morphology than *Helminthosphaeriaceae*. In this paper, we also report the occurrence of *Neoleptosporella camporesiana* on dead leaves of *Dracaena* sp.

*Neoleptosporella* Phukhmams. & K.D. Hyde

Phukhamsakda [47] introduced *Neoleptosporella* with *Neoleptosporella clematidis* as the type under Chaetosphaeriales, genera *incertae sedis*. Later, *Neoleptosporellaceae* was introduced to accommodate the genus *Neoleptosporella*. The genus is characterized by subglobose to depressed globose ascomata, shiny black, visible ostioles and immersed beneath a small clypeus. Ostioles with periphyses. Peridium is dark brown to black cells of textura angularis, hyaline, branched and septate paraphyses, broad, cylindrical and long-pedicellate asci, fusiform and aseptate ascospores [46,47].

#### *Neoleptosporella camporesiana* R.H. Perera & K.D. Hyde, in Hyde et al. [21], Fungal Diversity 100: 219 (2020)

Index Fungorum number: IF553466 Facesoffungi number: FoF06926.

Saprobic on dead leaves of *Dracaena* sp. Asexual morph: Not observed. Sexual morph: Appearing as a black spot raised dome-shaped, with a central short papilla. Ascomata 250–350 × 500–600 μm ($\bar{x}$ = 300 × 550 μm, *n =* 20), solitary or aggregated, immersed beneath small clypeus, subglobose to depressed globose. Hamathecium 10–15 μm wide ($\bar{x}$ = 13 μm, *n =* 10), hyaline, branched, septate, paraphyses. Asci 150–200 × 10–15 μm ($\bar{x}$ = 180 × 13 μm, *n =* 20), 8-spored, unitunicate, cylindrical, short pedicellate, apex rounded with a wedge-shaped, J- apical ring. Ascospores 40–60 × 2.5–4 μm ($\bar{x}$ = 50 × 3.25 μm, *n =* 20), fasciculate, parallel becoming spiral at maturity, filiform, straight or curved, hyaline, rounded at the apex, smooth-walled, guttulate, without appendages.

Material examined: Thailand, Tak Province, on dead leaves of *Dracaena* sp., 21 August 2019, Napalai Chaiwan, Tak33 (MFLUCC 22-0130, new host record).

GenBank accession number: ITS: ON166827, LSU: ON159749.

**Figure 1:**
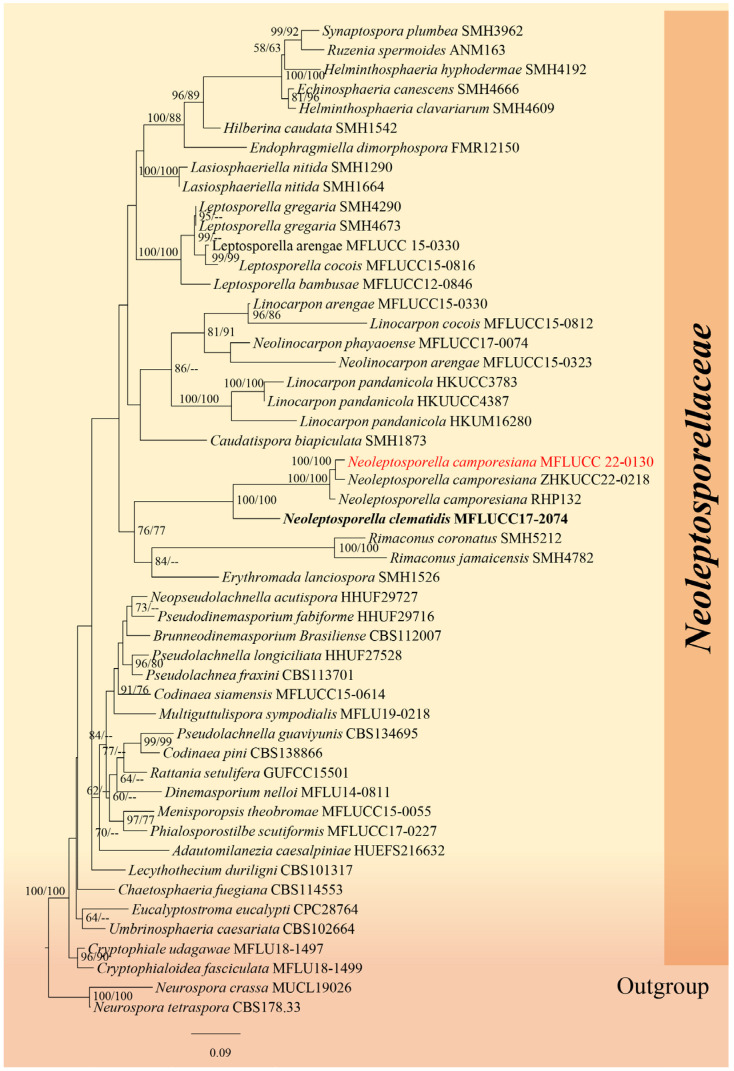
Phylogenetic tree of the family *Neoleptosporellaceae* constructed from RAxML analyses based on a two-gene dataset (LSU and ITS). Support values are on the branches (MP)/(ML). RAxML bootstrap support values ≥ 60% (ML = Left; MP = Right) are shown at nodes. The new sequences in this study are in red, the bold text is type strain.

Notes: In the phylogenetic analysis of *Neoleptosporellaceae* taxa, our species (MFLU 22–0130) was nested with *N. camporesiana* (MFLUCC 15-1016, type species) with 100% in ML and 100% in MP (Figure 1). Nucleotide comparison of ITS of our isolate and the type strain of *N. camporesiana*, and *N. yunnanensis* showed 16 bp and 10 bp differences, respectively. The LSU of our isolate and the type strain of *N. camporesiana* and *N. yunnanensis* showed 16 bp and 18 bp differences, respectively. Our species has the smaller ascomata ($\bar{x}$ = 300 × 550 μm) than *N. camporesiana* MFLUCC 15-1016, type species ($\bar{x}$ = 250 × 800 μm) but similar asci size. Our species showed a smaller ascospore ($\bar{x}$ = 50 × 3.25 μm) than *N. camporesiana* type species ($\bar{x}$ = 126 × 3 μm).

**Figure 2:**
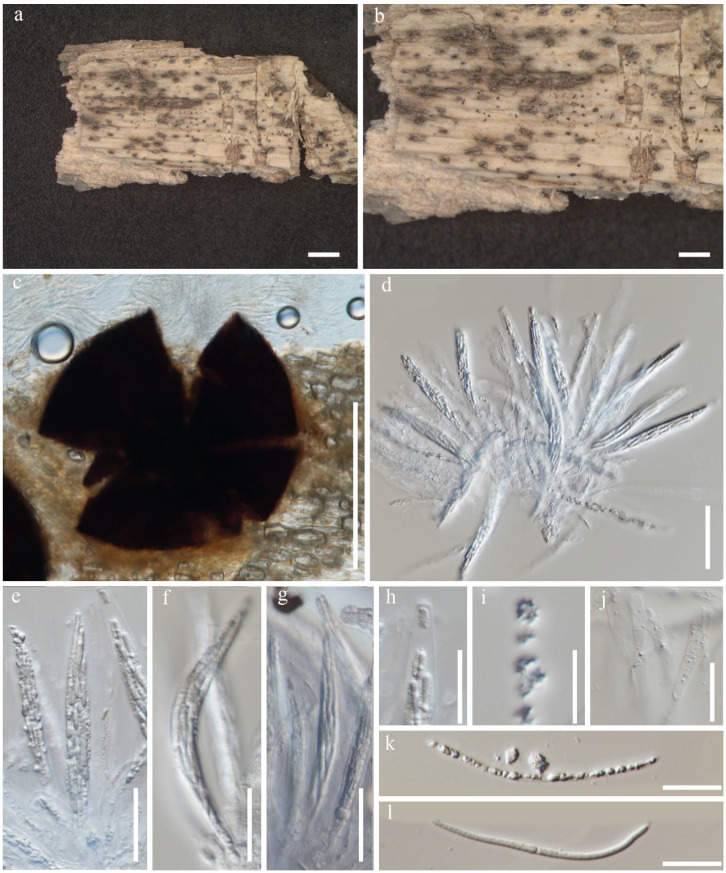
*Neoleptosporella camporesiana* (MFLU 22-0130). (**a**) Herbarium material; (**b**) ascomata on host substrate; (**c**) a squashed ascoma; (**d**–**g**) Asci; (**h**,**i**) tip of ascus; (**k**,**l**) ascospores; (**j**) paraphyses. Scale bars: (**a**,**b**) = 1000 μm; (**c**) = 50 μm; (**d**–**g**) = 20 μm; (**h**–**l**) = 10 μm.

Hence, we report *N. camporesiana* (MFLU 22-0130) as a new host record-based morphology and available phylogenetic analysis support. Our strain was found on *Dracaena* sp. (Tak Province, Thailand), whereas the holotype of *N. camporesiana* was found on a dead branch of an unidentified plant in Chiang Rai Province, Thailand. Based on morphological similarity and phylogenetic evidence, we identified our new collection as a new host record of *Neoleptosporella camporesiana* (Figure 2). We could not obtain culture as the spores of this species did not germinate. The other remaining member of this genus is the type species, *Neoleptosporella clematidis*, which was reported from *Clematis subumbellata* in Chiang Rai Province, Thailand [47]. A recent study reported the distribution and substrates of Neoleptosporella strain. In 2026, a new species *Neoleptosporella agapanthi* was described from Brazil on stalks of *Agapanthus praecox* (*Amaryllidaceae*), and *Neoleptosporella fusiformispora* (GZCC 20-0158, ex-type) species have been reported from China on decaying stems of unidentified grasses (Poaceae).

### 3.2. Glomerellales Chadef. ex Réblová, W. Gams & Seifert

Glomerellales has five families: *Australiascaceae*, *Glomerellaceae*, *Malaysiascaceae*, *Plectosphaerellaceae*, and *Reticulascaceae* [49]. Members of this order are known as pathogens, endophytes, or saprobes [29,50,51,52].

#### 3.2.1. *Glomerellaceae* Locq. ex Seifert & W. Gams

*Glomerellaceae* is a monotypic family that comprises only the genus *Colletotrichum* [21]. *Colletotrichum* species are known to be associated with various plants such as saprobes, pathogens, and endophytes. There are 16 significant species complexes and 15 individual species within *Colletotrichum* [29,53].

*Colletotrichum* Corda

*Colletotrichum* is known to exhibit diverse lifestyles, including endophytes, plant pathogens, and saprobes [54]. As a significant plant pathogenic genus, *Colletotrichum* displays considerable genetic variability, as highlighted by recent studies [29]. Bhujun [55] emphasized the necessity of species identification in this genus using polyphasic approaches. In this study, we describe one new species, document three new host records, and synonymize two existing species.

#### *Colletotrichum dracaenophilum* D.F. Farr & M.E. Palm, Mycol. Res. 110: 1401. 2006

Index Fungorum number: IF510231 Facesoffungi number: FoF10776.

Saprobic on dead leaves of *Dracaena* sp. Asexual morph: Conidiomata solitary or aggregate. Conidiomata, conidiophores and Setae abundant, arising from acervuli, dark brown to pale brown, smooth-walled to slightly verruculose, straight to slightly flexuous, formed directly on hyphae. Conidiophores hyaline to medium brown, smooth-walled, septate. Conidiogenous cells 9–12 × 3–6 μm, hyaline, smooth-walled, cylindrical, the upper part sometimes surrounded by a gelatinous sheath, 12–24 × 3–6 μm, opening 1–2 μm diam, collarette 0.5 μm long, periclinal thickening distinct. Conidia hyaline, smooth-walled, aseptate, cylindrical to oblong, straight to slightly curved, with both ends rounded or one end slightly acute, the apex rounded, the base rounded to truncate, 15–20 × 4–5 μm ($\bar{x}$ = 17.5 × 4.5 μm, *n =* 20), sometimes tapering to the base and sometimes slightly curved. Appressoria not formed. Sexual morph not observed.

Material examined: Thailand, Ratchaburi Province, Pak Tho district, on dead leaves of *Dracaena* sp., 2 September 2017, Saranyaphat Boonmee, SBRBR1 (MFLU 18-1858, living culture MFLUCC 18-0489).

GenBank accession number: ITS: OM988430.

**Figure 3:**
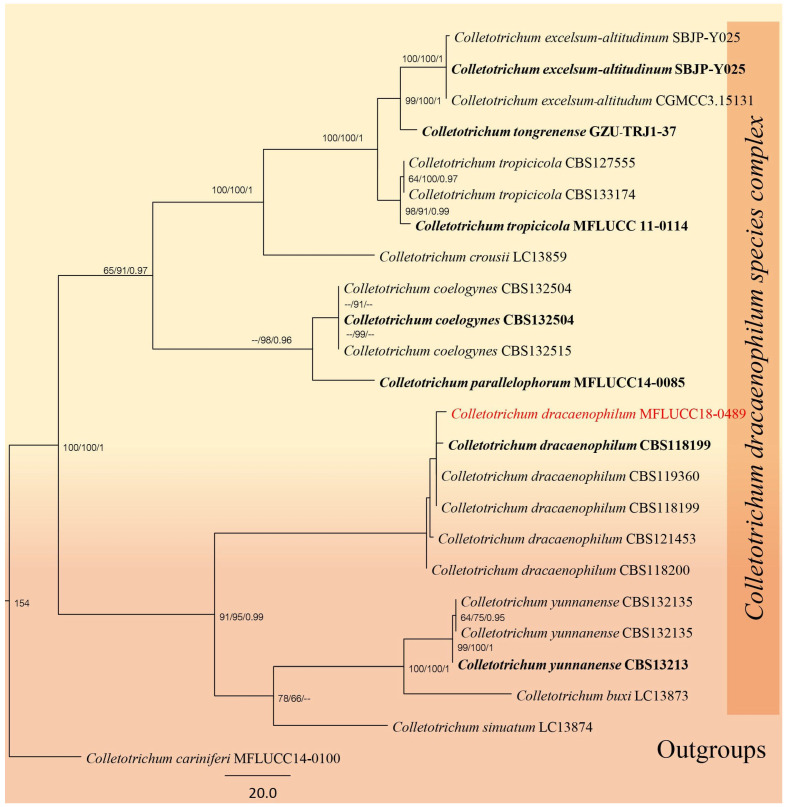
Phylogenetic tree of the *Colletotrichum dracaenophilum* species complex constructed from RAxML analyses based on a five-gene dataset (ITS, GAPDH, CHS, ACT and TUB). Support values are on the branches (MP)/(ML). RAxML bootstrap support values ≥ 60% (ML = Left; MP = Right) are shown at nodes. The new sequences in this study are in red, the bold text is type strain.

**Figure 4:**
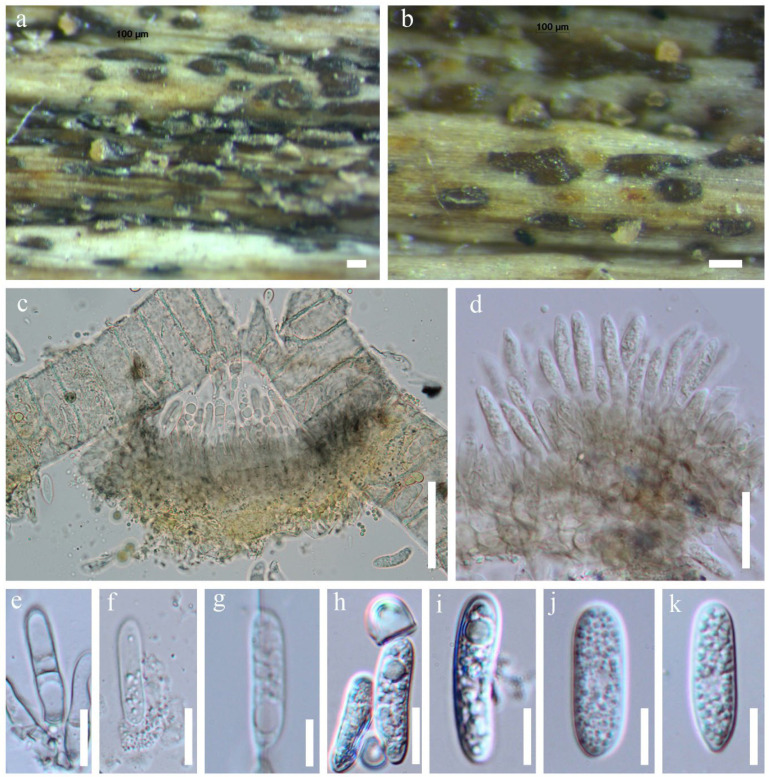
*Colletotrichum dracaenophilum* (MFLU 18-1858). (**a**,**b**) Fruiting body on dead leaf of *Dracaena* sp.; (**c**–**e**) Conidiogenous cells with conidia; (**f**–**k**) Conidia. Scale bars: (**a**,**b**) = 100 μm; (**c**) = 40 μm; (**d**) = 20 μm; (**e**–**k**) = 10 μm.

Notes: *Colletotrichum dracaenophilum*, initially described by Farr [56] as a stem pathogen of *Dracaena sanderiana* in California, USA, has also been reported on *D. sanderiana* in Australia, Bulgaria, China, Egypt, and Florida (USA), as well as on *D. braunii* in Brazil [56,57]. In our multi-loci phylogenetic analysis (Figure 3, Table 2), our strain clustered with *Colletotrichum dracaenophilum* strains [58]. Morphologically, the new isolate has conidiophores ($\bar{x}$ = 9–11 × 2–3 μm, *n =* 20) that are smaller than those of the type species ($\bar{x}$ = 10–16 × 5–6 μm) and conidia ($\bar{x}$ = 17.5 × 4.5 μm, *n =* 20) that are also smaller than the type species ($\bar{x}$ = 22.2 × 6.6 μm (Figure 4). The closest sequence matches in GenBank are with *C. dracaenophilum* strains included in this study (Figure 2). Therefore, our strain represents a new record of *C. dracaenophilum* from *Dracaena* sp. in Thailand.

#### *Colletotrichum gloeosporioides* (Penz.) Penz. & Sacc., Atti Inst. Veneto Sci. lett., ed Arti, Sér. 6 2(5): 670 (1884)

Synonyms: *Colletotrichum dimorphum* Z.F. Yu, in Yu, Jiang, Zheng, Zhang & Qiao, J. Fungi 8(2, no. 185): 14 (2022).

*Colletotrichum nanhauense* Z.F. Yu, in Yu, Jiang, Zheng, Zhang & Qiao, J. Fungi 8(2, no. 185): 17 (2022).

Notes: *Colletotrichum gloeosporioides* was first reported by Penzig [59] as *Vermicularia gloeosporioides* from *Citrus* in Italy. *Colletotrichum gloeosporioides* have been documented on many hosts and are commonly found as dominant endophytes in tropical herbaceous plants [60,61]. This species is also known to infect a variety of fruits, including almonds, avocados, apples, coffee, cranberries, guava, mango, strawberries, papaya, bananas, passion fruit, citrus, grapes, and cashews [62].

**Figure 5:**
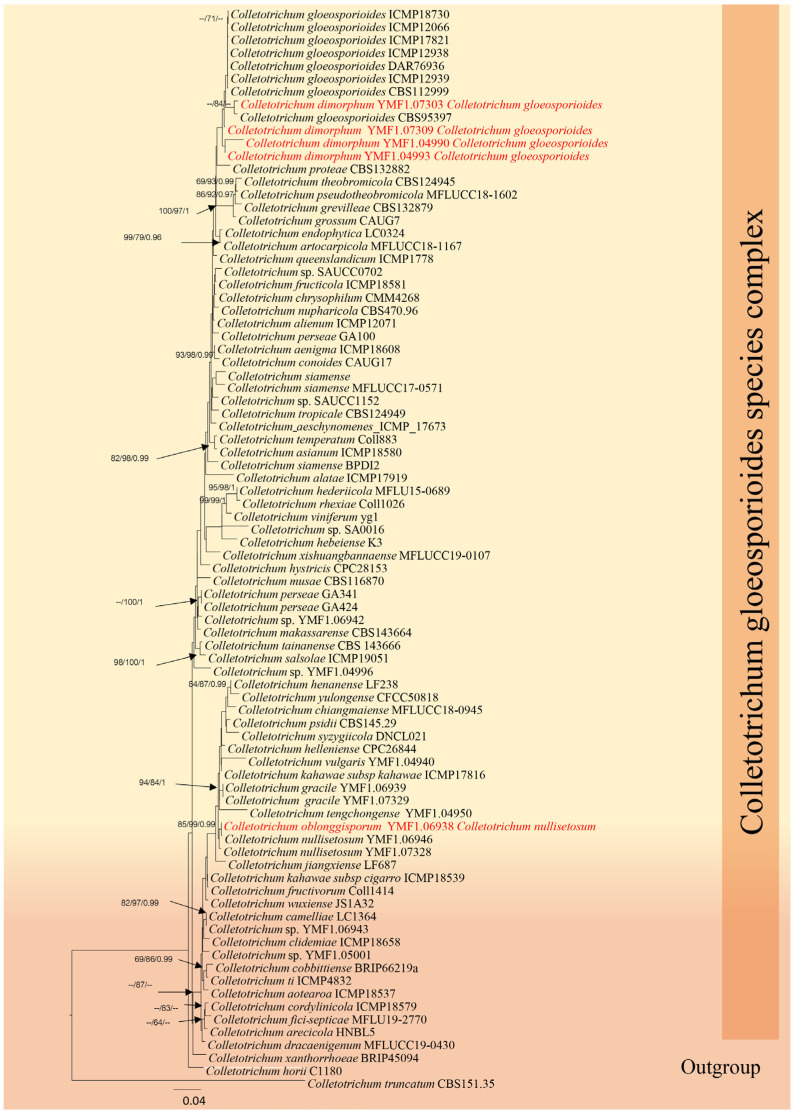
Phylogenetic tree of the *Colletotrichum gloeosporioides* species complex constructed from RAxML analyses based on a five-gene dataset (ITS, *GAPDH*, *CHS*, *ACT* and *TUB*). Support values are on the branches (MP)/(ML). RAxML bootstrap support values ≥ 60% (ML = Left; MP = Right) are shown at nodes. The new sequences in this study are in red.

In our phylogenetic analysis (Figure 5), we found that the newly identified species *Colletotrichum dimorphum* and *C. nanhauense*, introduced by Yu [63], clustered with *Colletotrichum gloeosporioides*. The morphology of these species closely resembles *C. gloeosporioides*; however, there can be morphological overlap. Therefore, we compared the sequence data of these two strains. A comparison of the ACT gene regions of *Colletotrichum dimorphum* (YMF1.07303) and *Colletotrichum gloeosporioides* (CBS95397) revealed 7 out of 298 (2.34%) nucleotide differences (Table 3). Based on the combined phylogenetic and morphological evidence, we synonymize *Colletotrichum dimorphum* (YMF 1.07309 and YMF1.07303) and *C. nanhuaense* (YMF 1.04990 and YMF1.04993) with *Colletotrichum gloeosporioides*.

*Colletotrichum nullisetosum* Váňová, Česká Mykol. 45(3): 128 (1991) Z.F. Yu, in Yu, Jiang, Zheng, Zhang & Qiao, Journal of Fungi 8(2, no. 185): 18 (2022).

Synonym: *Colletotrichum oblongisporum* Z.F. Yu, in Yu, Jiang, Zheng, Zhang & Qiao, J. Fungi 8(2, no. 185): 20 (2022).

Notes: *Colletotrichum oblongisporum* was introduced by Yu et al. (2022) [63]. In our phylogenetic analysis (Table 4), molecular comparisons showed that *Colletotrichum oblongisporum* clustered with *Colletotrichum nullisetosum*. Comparisons of morphological characteristics and gene sequences suggest that these species are conspecific. Therefore, we synonymize *Colletotrichum oblongisporum* (YMF1.06938) with *Colletotrichum nullisetosum* (YMF1.07328).

#### *Colletotrichum gigasporum* Rakotonir. & Munaut, in Rakotoniriana, Scauflaire, Rabemanantsoa, Urveg-Ratsimamanga, Corbisier, Quetin-Leclercq, Declerck & Munaut, Mycol. Progr. 12(2): 407 (2012) [2013]

Index Fungorum number: IF 800175; Facesoffungi number: FoF10777.

Saprobic on dead leaves of *Dracaena* sp. Sexual morph: Not observed. Asexual morph: Conidiomata acervular, conidiophores and setae formed on a cushion of roundish to angular brown cells. Setae pale to medium brown, smooth-walled to verruculose 1–3-septate, 50–150 μm long, base cylindrical, 3–5 μm. diam, tip acute to obtuse. Conidiophores pale brown, septate, branched. Conidiogenous cells pale brown, cylindrical to clavate, 15–25 × 4–8 μm, opening 1–2.5 μm diam. Conidia hyaline, aseptate, smooth-walled, cylindrical to slightly curved, both ends rounded, 20–30 × 3.5–6.5 μm.

Material examined—Thailand, Chiang Mai Province, Mae Tang District, on dead leaves of *Dracaena* sp., 16 February 2019, Napalai Chaiwan, MRC5 (MFLU 22-0150).

GenBank accession numbers: ITS: OM990842, *TUB2*: OP099906, *GAPDH*: OP099905.

**Figure 6:**
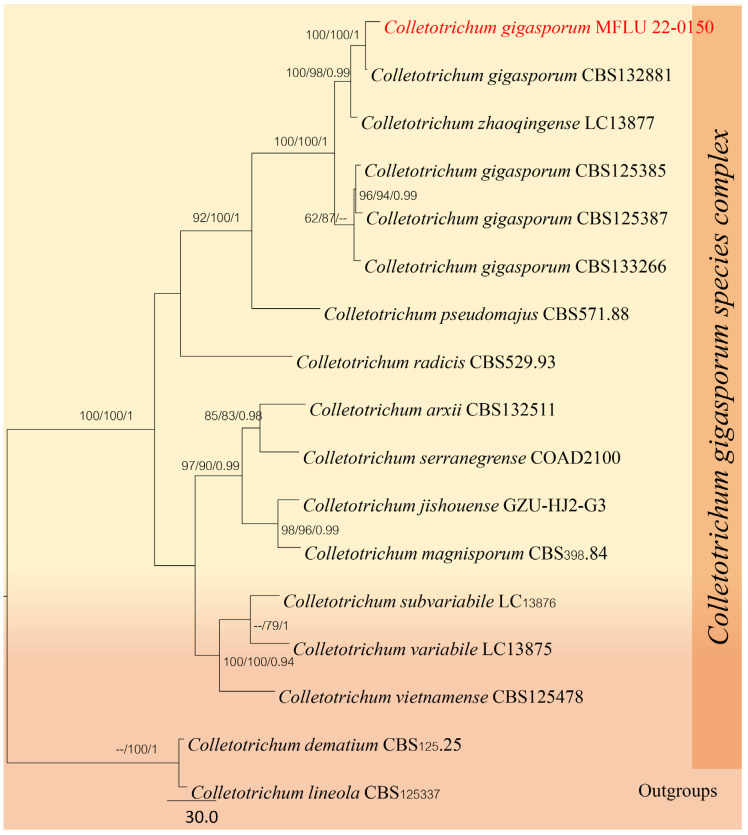
Phylogenetic tree of the *Colletotrichum gigasporum* species complex constructed from RAxML analyses based on a five-gene dataset (ITS, *GAPDH*, *CHS*, *ACT* and *TUB*). Support values are on the branches (MP)/(ML). RAxML bootstrap support values ≥ 60% (ML = Left, MP = Right) are shown at nodes. The new sequences in this study are in red.

**Figure 7:**
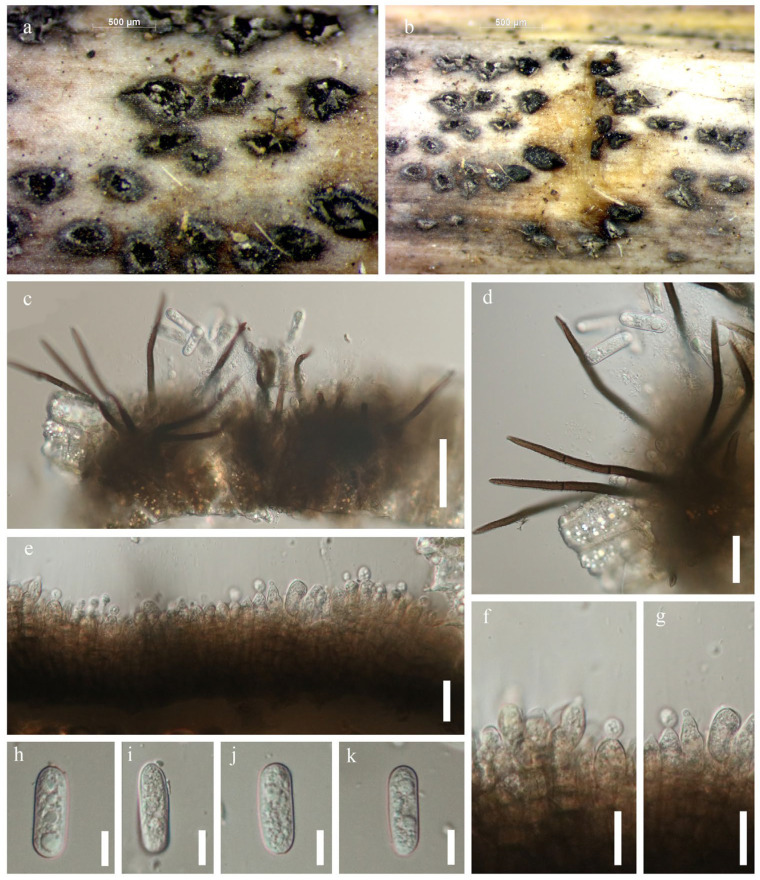
*Colletotrichum gigasporum* (MFLU 22-0150). (**a**,**b**) Fruiting body on dead leaf of *Dracaena* sp.; (**c**–**g**) Conidiophore; (**e**–**g**). Conidiogenous cells; (**h**–**k**) Conidia. Scale bars: (**a**,**b**) = 500 μm; (**c**) = 50 μm; (**d**–**g**) = 20 μm; (**h**–**k**) = 10 μm.

Notes: *Colletotrichum gigasporum* is distinguished by its larger conidia, as noted by Liu [4]. In their study, Liu [4] proposed synonymizing *C. thailandicum* with *C. gigasporum* based on ITS and TUB2 sequence data from phylogenetic analysis (Figure 6, Table 5), given *that C. gigasporum* was described earlier than *C. thailandicum*. *Colletotrichum gigasporum* is typically found on plants as either endophytes or pathogens, and some strains have even been isolated from human tissue [64]. However, we could not obtain a culture for this study as the spores of this species failed to germinate. Our strain clustered with *C. gigasporum* in multi-loci analyses (Figure 7), marking the first report of *C. gigasporum* on *Dracaena* sp. from Thailand.

In our phylogenetic analysis, *C. zhaoqingense* (LC13877) clustered with *C. gigasporum*. The gene combination order used in the original description of *C. zhaoqingense* differs from the order we used (Table 5), which aligns with the methodology of Jayawardena et al. (2021) [29]. Given this clustering, *C. zhaoqingense* (LC13877) should be synonymized with *C. gigasporum*. Therefore, we recommend that future studies consider synonymizing *C. zhaoqingense* under *C. gigasporum*, pending further evidence.

#### *Colletotrichum truncatum* (Schwein.) Andrus & W.D. Moore, Phytopathology 25: 122 (1935)

Index Fungorum number: IF280780 Facesoffungi number: FoF03827.

Saprobic on dead leaves of *Dracaena* sp. Sexual morph: Not observed. Asexual morph: Setae abundant, pale to medium brown, smooth walled, 1–5 septate, 100–200 μm ($\bar{x}$ = 150) μm (*n* = 10) long, base cylindrical, 4–10 μm ($\bar{x}$ = 7 μm, *n =* 20) diam., tip somewhat acute. Conidiophores hyaline, smooth-walled, simple, 10–20 μm ($\bar{x}$ = 15) μm (*n =* 20) long. Conidiogenous cells 1–2 μm × 2–3.5 μm ($\bar{x}$ = 1.5 μm × 2.75 μm, *n =* 10), hyaline, smooth-walled, cylindrical to slightly inflated, opening 1–2.5 μm ($\bar{x}$ = 1.7 μm, *n =* 20 diam). Collarette present, 0.4–1.1 μm ($\bar{x}$ = 0.8 μm, *n =* 20 μm width), periclinal thickening visible. Conidia 20–30 μm × 2–4 μm ($\bar{x}$ = 25 × 3 μm, *n =* 40), hyaline, smooth or verruculose, aseptate, curved, both sides gradually tapering towards the round to slightly acute apex and truncate base, guttulate.

Material examined: Thailand, Songkla Province, Hat Yai district, on dead leaves of *Dracaena* sp., 5 September 2018, Napalai Chaiwan, TNC9 (MFLU 22–0134).

GenBank accession number—ITS: OM991937, *ACT*: OP099907, *CHS*: OP099908, *GAPDH*: OP099909.

**Figure 8:**
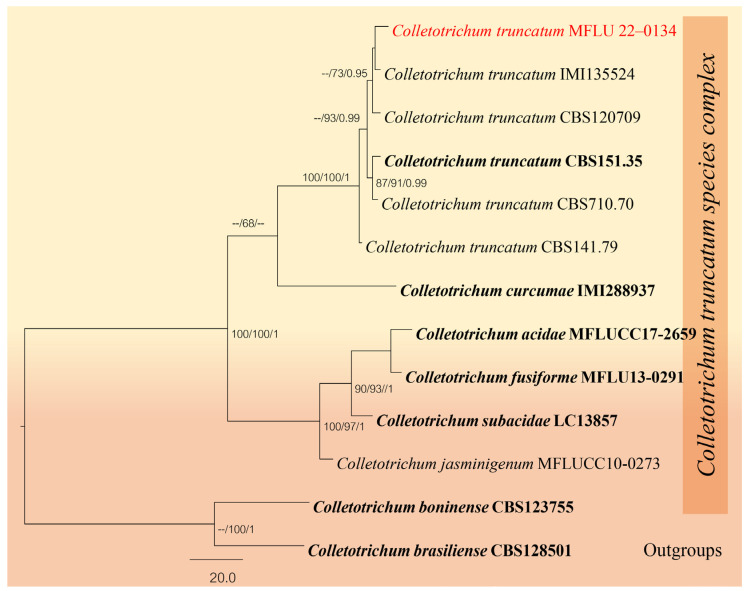
Phylogenetic tree of the *Colletotrichum truncatum* species complex constructed from RAxML analyses based on a four-gene dataset (ITS, *GAPDH*, *CHS* and *ACT*). Support values are on the branches (MP)/(ML). RAxML bootstrap support values ≥ 60% (ML = Left; MP = Right) are shown at nodes. The new sequences in this study are in red, the bold text is type strain.

**Figure 9:**
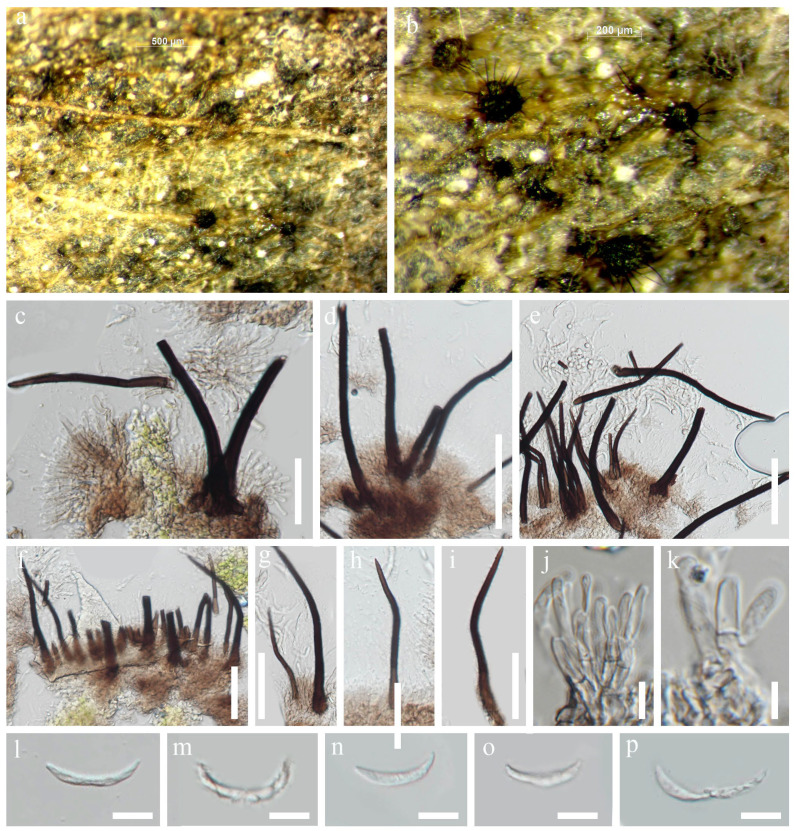
*Colletotrichum truncatum* (MFLU 22-0134). (**a**,**b**) Fruiting body on dead leaf of *Dracaena* sp.; (**c**–**i**) Conidiophore; (**j**,**k**) Conidiogenous cells; (**l**–**p**) Conidia. Scale bars: (**a**) = 500 μm; (**b**) = 200 μm; (**c**–**i**) = 20 μm; (**j**,**k**) = 10 μm; (**l**–**p**) = 5 μm.

Notes: *Colletotrichum truncatum* is known to cause anthracnose diseases in various hosts (Damm et al. 2009) [65]. In our phylogenetic analysis (Figure 8), our strain clustered with the ex-epitype of *C. truncatum*. Therefore, we report our strain as a new record of *C. truncatum* on *Dracaena* sp. in Thailand (Figure 9).

### 3.3. Malaysiascaceae Tibpromma & K.D. Hyde

Malaysiascaceae was introduced by Tibpromma [66] to include the monotypic genus *Malaysiasca*. Hyde [46] recognized *Malaysiasca* as the sole genus within Malaysiascaceae. Members of this family are reported as saprobes exclusively in Asia, commonly found on dead or decaying leaves and wood in terrestrial habitats [66,67].

*Malaysiasca* Crous & M.J. Wingf.

The sexual morph of this genus features perithecial ascomata with ellipsoid to oblong asci and hyaline, 1-septate ascospores. The asexual morph produces a fascicle of long, sub-cylindrical, unbranched, macronematous conidiophores that are erect, flexuous, pale brown, and smooth-walled. These conidiophores generate ellipsoidal to cylindrical-ellipsoid, somewhat clavate conidia with rounded apices; the conidia are aseptate, hyaline, solitary, smooth-walled, guttulate, and produced in a slimy mass [66,67]. Conidiogenous cells holoblastic, phialidic, hyaline to pale brown, smooth-walled, arising from the inner layers of the ascomatal wall or from short conidiophores; cylindrical to ampulliform or lageniform, discrete or integrated. In this study, we isolated seven strains, all identified as *Malaysiasca phaii*.

*Malaysiasca phaii* Crous & M.J. Wingf., in Crous et al. [67], Persoonia 36: 373 (2016).

Index Fungorum number: IF817045; Facesoffungi number: FoF04538.

Saprobic on the dead leaves of *Dracaena* sp. Sexual morph: Not observed. Asexual morph: Hyphomycetous. Conidiophores 150–220 × 4–5 μm ($\bar{x}$ = 185 × 4.5 μm, *n =* 20), subcylindrical, unbranched, macronematous, erect, flexuous, thick-walled, round at the apex, guttulate, 5–7-septate, pale brown to dark brown, becoming somewhat pale brown towards slightly tapered apex, smooth-walled. Conidiogenous cells 10–30 × 4–7 μm ($\bar{x}$ = 20 × 5.5 μm, *n =* 20), terminal, enteroblastic, phialidic, subcylindrical, subhyaline. Conidia 20–30 × 4–8 μm ($\bar{x}$ = 25 × 6 μm, *n =* 20), solitary, cylindric-ellipsoid, rounded at apex, aseptate, hyaline, smooth-walled, guttulate, produced in a slimy mass.

Material examined: Thailand, Kanchanaburi Province, Sangkhlaburi District, on dead leaves of *Dracaena* sp., 27 June 2019, Napalai Chaiwan, KAN1 (MFLU 22-0128), KLV5 (MFLU 22-0132), Tak Province, Lumpang District, on dead leaves of *Dracaena* sp., 21 August 2019, Napalai Chaiwan Tak1 (MFLU 22-0129), Tak3 (MFLU 22-013), Taksamngo1 (MFLU 22-0149), Krabi Province, on dead leaves of *Dracaena* sp., 19 December 2018, Napalai Chaiwan Krabi3 (MFLU 22-0131), Nakhon Si Thammarat Province, Cha-uat District, on dead leaves of *Dracaena* sp., 19 December 2018, Napalai Chaiwan NSW6 (MFLU 22-0133).

GenBank accession numbers: MFLU 22-0128 LSU: ON329745; MFLU 22-0129; LSU: ON000539; ITS: ON000540; MFLU 22-0130; LSU: ON000544; ITS: ON000543; MFLU 22-0131; LSU: ON000547; ITS: ON000545; MFLU 22-0132; LSU: ON000204; ITS: OM981248; MFLU 22-0133; LSU: ON000541; ITS: ON000542; MFLU 22-0149; LSU: PV746192.

**Figure 10:**
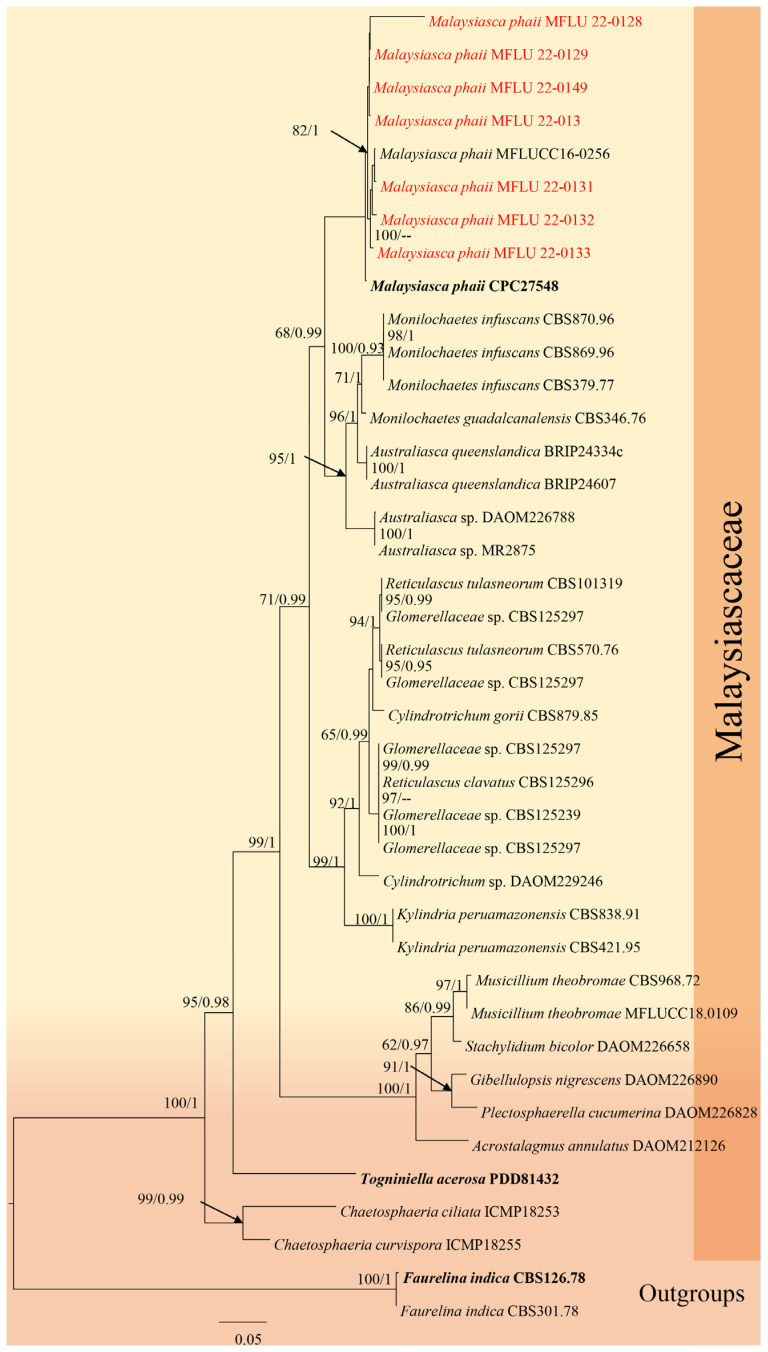
Phylogenetic tree of the *Malaysiasca* constructed from RAxML analyses based on a two-gene dataset (LSU and ITS). Support values are on the branches (MP)/(ML). RAxML bootstrap support values ≥ 60% (ML = Left; MP = Right) are shown at nodes. The new sequences in this study are in red, the bold text is type strain.

**Figure 11:**
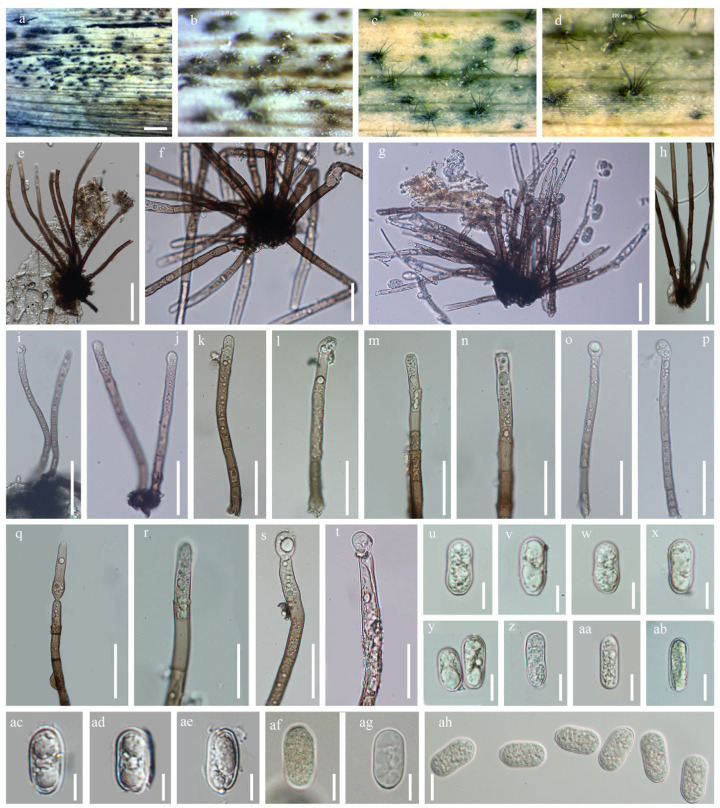
*Malaysiasca phaii* (MFLU 22-0128). (**a**–**d**) Colonies on dead leaf of *Dracaena* sp.; (**e**–**h**) Conidiophores; (**i**–**t**) Conidiophores, conidiogenous cells, and conidia; (**u**–**ah**) Conidia. Scale bars: (**a**) = 1000 μm; (**b**–**d**) = 200 μm; (**e**–**h**) = 100 μm; (**i**–**t**) = 50 μm; (**u**–**ah**) = 10 μm.

Notes: *Malaysiasca phaii* was introduced by Crous [67] and was initially collected in Malaysia. This is the only species in the family and has been reported from the leaves of *Phaius reflexipetalus* [67] and *Freycinetia javanica* [66]. In our phylogenetic analysis, the Thai strains from this study clustered with *M. phaii* (Figure 10). Although *M. phaii* has been previously reported on *Freycinetia javanica* in Thailand [37], this represents the second report of this species from Thailand. Additionally, this study provides the first record of *M. phaii* from *Dracaena* sp. (Figure 11).

Various Xylariales have been isolated from both saprobic environments and as plant pathogens [68]. The order is characterized by well-developed stromata, perithecial ascomata with thick walls, and eight-spored unitunicate asci with a J+ apical apparatus. Ascospores are typically pigmented, may have germ pores or slits, and can be either transversely septate or surrounded by a mucilaginous sheath [69,70]. The asexual morphs of Xylariales feature holoblastic conidial production [71,72].

#### 3.3.1. Zygosporiaceae J.F. Li, Phook. & K.D. Hyde

Hyde [46] recognized a single genus, *Zygosporium*, within the family *Zygosporiaceae*. Taxa in *Zygosporiaceae* are commonly found as saprobes on various plants and have a widespread distribution, ranging from temperate to tropical regions [21,73]. *Zygosporiaceae* is a lesser-known fungal family, and its taxonomic placement has been subject to revisions over time as molecular phylogenetics advanced [73,74].

*Zygosporium* Mont.

*Zygosporium* has been isolated as either saprobic or endophytic from a range of plants across temperate and tropical regions [75,76,77,78,79,80,81,82]. This genus is identified solely by its hyphomycetous asexual morphs, characterized by setiform conidiophores, darkly pigmented, incurved vesicular cells that produce 2–4 ampulliform conidiogenous cells, and single-celled conidia that are usually ellipsoid or globose, and can be smooth or variously ornamented [73]. In this study, we isolated three strains that belong to two new species.

##### *Zygosporium dracaenae* Chaiwan & Jayaward, sp. nov

Index Fungorum number: IF 371607; Facesoffungi number: FoF12774.

Saprobic on the dead leaves of *Dracaena* sp. Sexual morph: Not observed. Asexual morph: Hyphomycetous, Mycelium 2.5–4 μm wide, superficial on the substrate, composed of smooth, cylindrical, thin-walled, brown hyphae. Conidiophores setiform portion 60–100 µm long × 4–5.5 µm towards the base, 1–1.5 µm wide at the apical cell, macronematous, mononematous, consisting of a setiform portion with a vesicle borne on a short cell near its base, light brown to pale brown, thin-walled, smooth, solitary or in small groups. Conidiogenous cells 3–5 × 1–3 μm ($\bar{x}$ = 4 × 2 μm, *n =* 20), arise directly from the vesicular cell. Conidia 4–6 × 1–3 μm ($\bar{x}$ = 5 × 2 μm, *n =* 20), solitary, initially globose, subglobose, aseptate, smooth, subhyaline to brown, thick-walled.

Material examined: Thailand, Kanchanaburi Province, on dead leaves of *Dracaena* sp., 24 October 2018, Napalai Chaiwan, KAN12, (holotype MFLU 22-0124).

GenBank accession numbers: ITS: OM914646.

**Figure 12:**
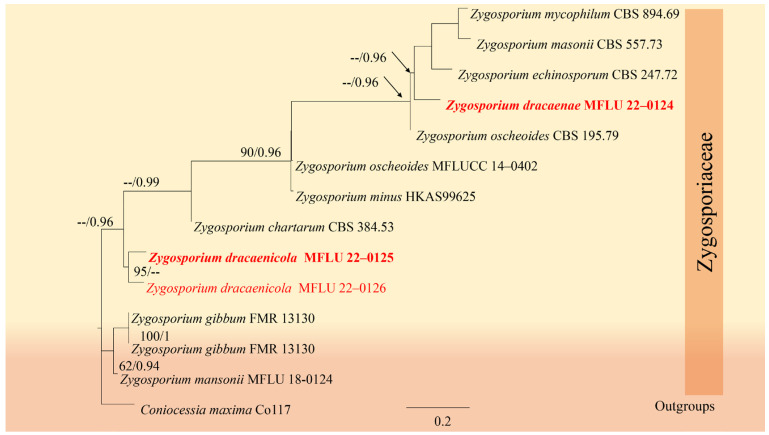
Phylogenetic tree of the *Zygosporaceae* constructed from RAxML analyses based on a two-gene dataset (LSU and ITS). Support values are on the branches (MP)/(ML). RAxML bootstrap support values ≥ 60% (ML = Left, MP = Right) are shown at nodes. The new sequences in this study are in red, the bold text is type strain.

**Figure 13:**
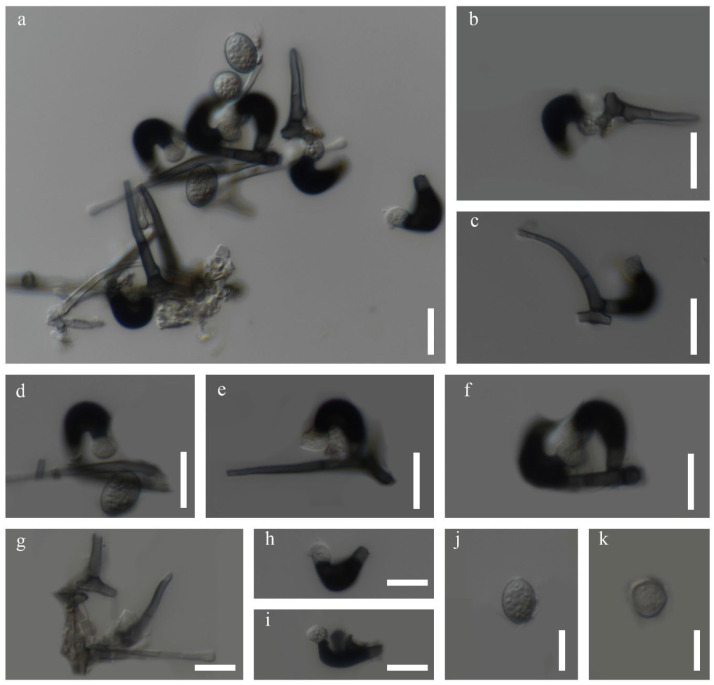
*Zygosporium dracaenae* (MFLU 22-0124 Holotype). (**a**,**b**,**g**) Conidiophores with vesicles; (**c**–**f**,**h**,**i**) Vesicles and conidiogenous cells; (**j**,**k**) conidia. Scale bars: (**a**–**k**) = 10 μm.

**Figure 14:**
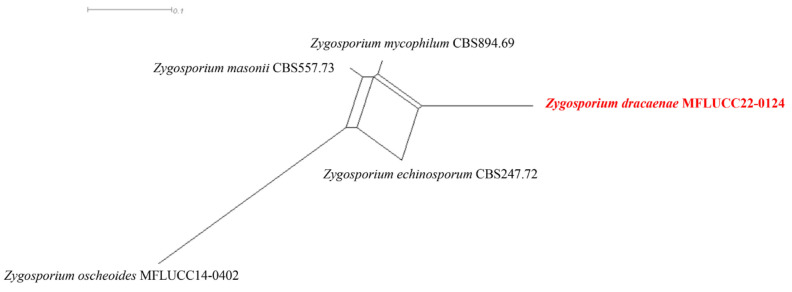
Results of the pairwise homoplasy index (PHI) test of *Zygosporium dracaenae* and closely related species using both LogDet transformation and split decomposition. PHI test results (Φw) < 0.05 indicate significant recombination within the dataset. The new taxon is in bold red. *p* = 1.

Notes: Based on our multi-loci analyses, our isolate grouped in a sister clade with *Zygosporium echinosporum*, *Z. masonii*, *Z. mycophilum*, and *Z. oscheoides* (Figure 12, Table 6). Blastn search results indicated that the ITS sequences of our isolate are 89.14% identical to those of *Z. oscheoides* (MFLUCC 14-0402), which was found on *Ptychosperma macarthurii* (Arecaceae). Morphologically, our isolate resembles *Z. pandanicola*, which is distinguished by vesicular cells produced from the sides of setiform conidiophores, smooth, acute or narrowly clavate setiform conidiophores, and spherical to globose, hyaline to pale brown conidia that are smooth or minutely verruculose [76] (Figure 13). However, we could not obtain a culture as the spores failed to germinate. Therefore, we designate our isolate as the new species *Zygosporium dracaenae* from *Dracaena* sp. in Thailand (Figure 14).

##### *Zygosporium dracaenicola* Chaiwan & Jayaward, sp. nov

Index Fungorum number: IF 325704; Facesoffungi number: FoF12775.

Saprobic on the dead leaves of *Dracaena* sp. Asexual morph: Hyphomycetous, Mycelium 2.5–4 μm wide, superficial, in substrate superficial, cylindrical. Setae and setiform conidiophores 60–100 × 4–5.5 μm towards the base, 1–1.5 μm wide at the apical cell subhyaline, smooth, narrowly clavate, septa, erect, straight, curved or flexuous, tapered from the base, narrowing towards the apex, smooth, 3–4-septate, brown except for the pale apical cell, thickened walls and obtuse, upper section of the setiform conidiophore sterile, first cell giving rise to a single vesicular conidiophore, basal cell concolorous with the lower parts of the setae, branching in two directions becoming part of the mycelium and connecting the conidiophores. Vesicular conidiophores 5–8 × 4.5–5 μm, arising on the side of the first cell of the setiform conidiophore, comprise three cell types; stalk cell cylindrical, brown, thick-walled, smooth; vesicular cell dark brown to black, thick-walled, smooth, swollen, upwardly curved, inner curvature paler than the rest of the cell, 15–16 × 8–9 μm. Conidiogenous cells 4–6 × 1–3 μm ($\bar{x}$ = 5 × 2 μm, *n =* 20), arise directly from the vesicular cell, monoblastic, discrete, determinate, swollen, ampulliform, upwardly curved, smooth, pale brown, apex obtuse. Conidia 3.5–7.5 μm, solitary, dry, initially globose, later become broadly ellipsoid, aseptate, smooth, subhyaline to brown, thick-walled. Sexual morph: Not observed.

Material examined: Thailand, Nakhon Si Thammarat Province, on dead leaves of *Dracaena* sp., 19 December 2018, Napalai Chaiwan, Nakhon11, (holotype MFLU 22-0125). 23 August 2019, TAK province, Napalai Chaiwan, Umpang 3 (MFLU 22–0126).

GenBank accession numbers: MFLU 22–0125 LSU:OM914645; LSU:OM914644; MFLU 22–0126 LSU:OM914645.

**Figure 15:**
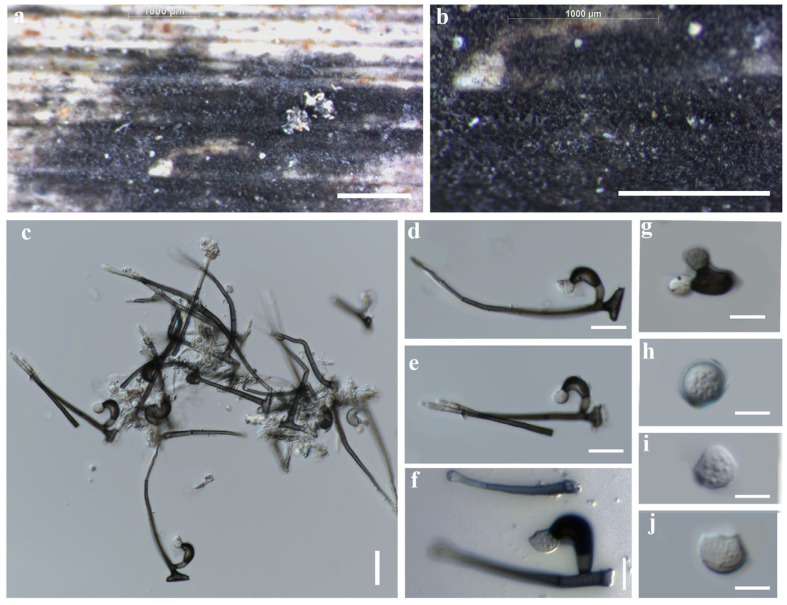
*Zygosporium dracaenicola* (MFLU 22-0125 Holotype). (**a**,**b**) Hyphomycete on dead leaf of *Dracaena* sp. (**c**–**g**) Setiform conidiophores with attached vesicular conidiophores. (**h**–**j**) Vesicular conidiophores with two conidiogenous cells and conidia. Scale bars: (**a**,**b**) = 1000 µm. (**c**) = 20 µm. (**d**–**j**) = 10 µm.

**Figure 16:**
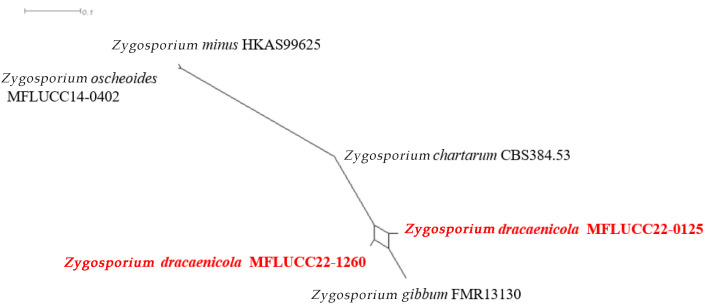
Results of the pairwise homoplasy index (PHI) test of *Zygosporium dracaenicola* closely related species using both LogDet transformation and split decomposition. PHI test results (Φw) < 0.05 indicate significant recombination within the dataset. The new taxon is in bold red. *p* = 0.9999.

Notes: In this study, we describe and illustrate the morphology of *Z. dracaenicola* and provide sequence data, establishing it as a new species on *Dracaena* sp. from Thailand (Figure 15 and Figure 16). Based on morphological comparisons with currently recognized species, our strains are more similar to *Z. chartarum*, *Z. minus* and *Z. oscheoides*, particularly in features such as vesicular conidiophores with three conidiogenous cells and a short, sterile apical cell, and ellipsoid, hyaline to pale brown conidia that are smooth or verruculose (Figure 15). However, these strains differ in size: the setae are smaller (30–70 μm long versus 60–100 μm long) and the conidia are larger (7–12 μm long) compared to *Z. dracaenicola* (3.5–7.5 μm long).

## 4. Discussion

In this study, we have significantly advanced the understanding of fungal diversity associated with *Dracaena* in Thailand [16,17,18]. We introduced two new species and five new host and geographical records, a substantial contribution to the field. Our paper emphasizes the value of integrating traditional and molecular methods in fungal taxonomy and ecology. We conducted our research across seven provinces in Thailand (Chiang Mai, Kanchanaburi, Krabi, Nakhon Si Thammarat, Ratchaburi, Songkhla, and Tak), where we collected fungal specimens associated with *Dracaena*. These specimens were identified through a thorough morphological examination supported by multi-loci phylogenetic analyses. Our collections, which include isolated cultures and directly sequenced fruiting structures, represent eight taxa across four Sordariomycetes families [21].

The research by Thongkantha [14] was a pioneering effort to explore fungal saprobes and pathogens associated with *Dracaena lourieri* in Thailand. They noted that only a portion of the taxa identified from *Dracaena* were found on both bait and natural leaves, with their descriptions primarily relying on phylogenetic analyses. This approach highlighted the importance of phylogenetic placement using multi-loci analysis of rDNA sequences. However, the morphology of these fungi has not been thoroughly examined, creating a gap in integrating morphological characteristics into a comprehensive taxonomic framework. This underscores the need for further research in this area, particularly in providing detailed morphological descriptions and enhancing taxonomic clarity for some species [17,18].

Our study reveals an intriguing overlap in fungal species reported on *Dracaena* with those identified in earlier research, underscoring a reassuring continuity in the fungal diversity associated with *Dracaena* in Thailand. Importantly, our research introduces two new species of *Zygosporium* (*Z. dracaenae* and *Z. dracaenicola*) specific to *Dracaena* in Thailand, thereby enhancing the known diversity within this genus. Additionally, our study provides the first report on *Dracaena* in Thailand [78], further advancing the understanding of fungi associated with these plants.

Our findings significantly advance our understanding of the fungi associated with *Dracaena* in Thailand, filling a crucial knowledge gap that is invaluable for future ecological and biodiversity research. These advancements have significant implications for the field of mycology, underscoring the need for ongoing exploration in this field [46]. Further investigation into the microfungi associated with *Dracaena* is essential to elucidate their impacts, epidemiology, and the secondary metabolites they produce. This research is not just a step forward but a significant contribution to the field of mycology, with far-reaching implications for our understanding of fungal diversity and its ecological roles.

## 5. Conclusions

In this study, we investigated and recorded seven taxa associated with *Dracaena* plants, including two new species and five new host records. The results of this study reveal that there is a diverse fungal community found on *Dracaena* throughout Thailand.

## Figures and Tables

**Table 1:** Details of the gene loci sequences and the primers used in this study.

Genes/Loci	PCR Primers (Forward/Reverse)	References for Primer
Actin (*ACT*)	(*ACT512F/ACT783R*)	[31]
Chitin synthase (*CHS-1*)	(*CHS79F/CHS345R*)	[31]
Glyceraldehyde-3-phosphate dehydrogenase (*GAPDH*)	(*GDF/GDR*)	[32]
Internal transcribed spacer 1, 5.8S ribosomal RNA (ITS)	(ITS4/ITS5)	[33]
28S ribosomal RNA (LSU)	(LR0R/LR5)	[34]
Beta-tubulin (*TUB2*)	(*Bt2a/Bt2b*)	[35]

**Table 2:** Comparison of gene characteristics for ITS regions among *Colletotrichum dracaenophilum* strains.

Taxa	ITS Base Pair Position		
	1	2	3
*C. dracaenophilum* (MFLUCC 18-0489)	G	A	G
*C. dracaenophilum* (CBS118119)	A	G	A
*C. dracaenophilum* (CBS121453)	A	G	A
*C. dracaenophilum* (CBS119360)	A	G	A
*C. dracaenophilum* (CBS118200)	A	G	A

**Table 3:** Comparison of gene characteristics for ITS, *TUB, GAPDH*, and *ACT* regions among *Colletotrichum gloeosporioides* (CBS95397), *C. dimorphum* (YMF1.07303), and *C. nanhuaense* (YMF1.04993).

Taxa	ITS	*TUB*		*GAPDH*						
	46	22	322	114	158	173	214	233	249	298
*C. gloeospoioides* (CBS95397)	A	C	C	G	G	G	G	C	G	T
*C. dimorphum* YMF1.07303	-	T	T	-	-	-	-	-	-	C
*C. nanhauense* YMF1.04993	T	-	-	A	T	C	C	T	A	C
Taxa	*ACT*									
	120	269	270	273	274	280				
*C. gloeospoioides* (CBS95397)	T	G	A	T	G	C				
*C. dimorphum* YMF1.07303	C	T	G	A	T	T				
*C. nanhauense* YMF1.04993	-	-	-	-	-	-				

**Table 4:** Comparison of gene characteristics for the ITS, *TUB*, *GAPDH*, and *ACT* regions between *Colletotrichum nullisetosum* (YMF1.07328) and *Colletotrichum oblongisporum* (YMF1.06938).

	ITS	*TUB*						*GAPDH*	*ACT*	*CHS*
		377	412	451	481	564	702			
*C. nullisetosum* (YMF1.07328)	No sequence to compare	C	C	G	A	C	T	No difference	No sequence to compare	
*C. oblongisporum* (YMF1.06938)		T	A	T	C	A	C		No sequence to compare	No sequence to compare

**Table 5:** Gene character comparisons of ITS, *TUB, GAPDH*, and *CHS* regions between the *C. gigasporum* (CBS132881) and *C. zhaoqingense* (LC13877).

	ITS		*TUB*				*GAPDH*							*CHS*	
	521	561	151	162	318	363	69	144	163	189	231	238	262	1	301
*Colletotrichum gigasporum* (CBS132881)	C	T	T	T	T	A	C	A	T	G	C	G	G	-	-
*Colletotrichum zhaoqingense* (LC13877)	T	C	C	G	C	C	T	C	C	A	T	A	A	T	A

**Table 6:** Comparison of gene characteristics for ITS regions among *Zygosporium dracaenae, Z. echinosporum, Z. masonii, Z. mycophilum*, and *Z. oscheoides*.

	ITS																																									
	148	149	150	157	159	160	186	187	188	189	197	218	248	249	250	254	265	267	277	282	354	360	369	402	410	425	457	465	468	484	506	511	525	529	531	549	576	594	605	609	616	644
*Z. dracaenae*	C	C	T	A	C	G	C	G	A	C	C	C	A	T	C	G	A	T	A	T	A	A	A	G	T	A	C	C	G	C	G	C	C	A	T	T	C	T	C	A	A	C
*Z. echinosporum*	C	A	C	C	G	C	G	G	C	C	T	T	T	T	C	G	A	C	A	C	G	T	G	A	T	A	C	G	T	C	C	C	T	G	T	G	C	C	C	A	T	G
*Z. masonii,*	T	C	G	C	C	T	A	G	C	C	C	T	A	C	A	T	T	A	T	T	G	T	G	A	C	G	T	A	T	A	C	T	T	T	C	A	T	C	A	C	T	G
*Z. mycophilum,* and	C	C	C	C	G	G	G	C	C	T	C	C	A	T	C	G	A	C	A	T	G	T	G	A	T	A	C	C	G	C	G	A	C	A	T	T	C	T	C	C	A	G
*Z. oscheoides*	C	C	T	T	C	G	A	G	C	C	T	T	A	T	A	T	T	A	T	T	G	T	G	A	C	G	T	A	T	A	C	T	T	T	C	A	T	C	A	C	T	G

## Data Availability

All sequences generated in this study were submitted to GenBank.
